# Neuromolecular mechanisms related to reflex behaviour in *Aplysia* are affected by ocean acidification

**DOI:** 10.1098/rsos.240329

**Published:** 2024-06-12

**Authors:** Jade M. Sourisse, Celia Schunter

**Affiliations:** ^1^ The Swire Institute of Marine Science, School of Biological Sciences, The University of Hong Kong, Pokfulam Road, , Hong Kong

**Keywords:** pH, sea hare, carbon dioxide, climate change, brain, neuromolecular

## Abstract

While ocean acidification (OA) impacts the behaviour of marine organisms, the complexity of neurosystems makes linking behavioural impairments to environmental change difficult. Using a simple model, we exposed *Aplysia* to ambient or elevated CO_2_ conditions (approx. 1500 µatm) and tested how OA affected the neuromolecular response of the pleural–pedal ganglia and caused tail withdrawal reflex (TWR) impairment. Under OA, *Aplysia* relax their tails faster with increased sensorin-A expression, an inhibitor of mechanosensory neurons. We further investigate how OA affects habituation training output, which produced a ‘sensitization-like’ behaviour and affected vesicle transport and stress response gene expression, revealing an influence of OA on learning. Finally, gabazine did not restore normal behaviour and elicited little molecular response with OA, instead, vesicular transport and cellular signalling link other neurotransmitter processes with TWR impairment. Our study shows the effects of OA on neurological tissue parts that control for behaviour.

## 1. Introduction

Ocean acidification (OA) can have impacts on both calcifying and non-calcifying organisms’ survival and development [1,2], physiology [3,4] and behaviour [5,6]. Fish exhibit behavioural impairments among others in antipredator response [7–9] and learning [10,11]. However, invertebrates such as snails and crabs have also displayed behavioural impairments caused by OA [12–14]. Given that behaviour can significantly impact population dynamics [15–17], it is crucial to understand the drivers and underlying mechanisms to predict the trajectories of behavioural responses in a rapidly changing environment [18,19].

One proposed cause of behavioural impairments in a reduced pH environment is altered gamma-aminobutyric acid (GABA) neurotransmission [20]. Gamma-Aminobutyric Acid (GABA) is a major inhibitory neurotransmitter found across the animal kingdom [21–23] and GABA_A_ receptors are ion channels whose activation in normal conditions results in neuron inhibition. When the partial pressure of CO_2_ (pCO_2_) is elevated in seawater, the acidosis response of marine animals modifies internal ion gradients and the subsequent activation of GABA_A_ receptors results in neuron excitation [20]. This hypothesis was built on the fact that animals reared in elevated CO_2_ conditions had behavioural impairments at least partially restored when treated with the GABA_A_ receptor antagonist gabazine such as fish [24,25] but also invertebrates [13,26]. Changes in the expression of genes involved in GABAergic neurotransmission in response to OA have also been found [27–30], supporting that elevated pCO_2_ acts in neuronal cells at the molecular level, affecting pathways throughout the brain which in turn can produce impairments at the whole organism level. Nevertheless, the complexity of the vertebrate brain brings difficulties in understanding how molecular alterations inside neurons caused by environmental changes such as OA modify the course of information transmission across synapses and throughout regions of the nervous system, in turn driving behavioural changes. Furthermore, recent studies have shown that ligand-gated channels other than GABA receptors, such as glutamate, acetylcholine and dopamine-gated chloride (Cl^−^) channels, probably play a role in behavioural changes caused by OA in some invertebrates [31,32].

One way of linking environmental influence with nervous system functioning and its behavioural output is through the study of a simple and well-studied neuro system, such as that of the California sea hare (*Aplysia californica*). Contrary to vertebrate brains, the nervous system of *Aplysia* is composed of only 20 000 large neurons [33]. Several of its behaviours have been characterized in neural networks that link the nervous system and sensory organs to muscles [34,35]. As a result, it is possible to obtain the gene expression profile of specific nerve ganglia that control for specific behaviours. In addition, *Aplysia* compensates for acidosis by accumulating bicarbonate ions (HCO_3_^−^) similar to fish, crustaceans and cephalopods [36]. Since GABA also binds to HCO_3_^−^-permeable ionotropic GABA receptors in invertebrates [32], the hypothesis of OA-altered GABA neurotransmission causing behavioural impairments should be applicable in this model species. One suitable behaviour performed by *Aplysia* to test this hypothesis is the tail withdrawal reflex (TWR): during the TWR, the tail is withdrawn for protection after a tactile stimulus is applied to it [37,38]. It is a behaviour for which controlling neurons in the pleural–pedal ganglia have been extensively described [37,39,40], with neuron populations sensitive to several specific neurotransmitters such as glutamate [41], dopamine, FMRFamide [42] and GABA [43]. The TWR was also shown to be impaired by OA itself [36]. Finally, TWR can be involved in non-associative learning, notably reflex habituation [44], which is probably caused by a presynaptic mechanism preventing transmitters release [45]. Such learning processes through repeated eliciting of the behaviour may therefore also be altered by OA by interfering with transmitter release regulation.

**Figure 1.**
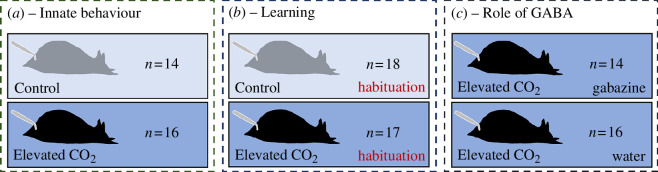
Experimental design for the study of *Aplysia*’s TWR response under elevated CO_2_ conditions. The TWR is elicited by a tap on the tail with a needle. In the learning experiment (*b*), animals received a habituation training and TWR responses were observed pre- and post-training. In the GABA experiment (*c*), animals were either momentarily exposed to gabazine or to control water. The number of biological replicates is indicated by the *n* value for each group.

We investigate the molecular processes affected by OA in the nervous system of the California sea hare (*A. californica*) as it performs a ‘simple’ behaviour. We observed the TWR response when animals were reared in either ambient (approx. 680 µatm) or elevated (approx. 1500 µatm) CO_2_ conditions. This further allows us to investigate whether OA influences learning experiences and the involvement of GABAergic neurotransmission in behavioural impairments. Our three experiments focused on (i) how the innate TWR behaviour changes with elevated CO_2_ and the underlying mechanisms in the nervous system, (ii) whether habituation training of the TWR is influenced by elevated CO_2_ and what corresponding neuromolecular changes take place in the reflex’s circuitry, and (iii) the potential role of GABAergic neurotransmission in the OA-induced behavioural changes of *Aplysia* (figure 1). We anticipate seeing a modification of the TWR associated with transcriptomic reprogramming notably involving genes associated with ligand-gated channels (such as GABA receptors) and genes playing a role in neurotransmitter release, both regarding the innate behaviour and after habituation training. Furthermore, we expect to see a behaviour reversal owing to gabazine. Together, these experiments pinpoint the molecular basis driving behavioural modifications when faced with future OA.

## 2. Methods

### 2.1. Experimental design

Adult sea hares weighing 52.8 ± 9.8 g were imported from the National Resource for *Aplysia* (University of Miami, USA) and acclimatized to their new environment for 3 days in ambient control conditions. After this acclimatization period, they were exposed to either ambient (pCO_2_ approx. 680 µatm; *n* = 36) or near-future CO_2_ conditions (pCO_2_ approx. 1500 µatm; *n *= 54) for 7–10 days at the University of Hong Kong. The elevated CO_2_ level was chosen following the RCP 8.5 scenario of the IPCC [46]. Animals were housed in recirculating systems composed of glass reservoirs (66 × 56 × 35.5 cm) and experimental tanks (41 × 21.5 × 37 cm), lidded, filled with natural seawater from Hong Kong Island shores circulating at 1.5 l min^−1^, with four to six individuals per tank (electronic supplementary material, figure S1). Two recirculating systems were used to house *Aplysia* exposed to elevated CO_2_ and one more was used to house *Aplysia* exposed to control conditions. Exposure of animals to elevated CO_2_ was created through bubbling a mix of air and CO_2_ gases at a rate of 15 l min^−1^, controlled by a PEGAS 4000 MF Gas Mixer (Columbus Instruments). To reach the desired pCO_2_, the mixing parameters were set as follows: air flow was at 15 000 cc min^−1^, CO_2_ flow (vCO_2_ in cc min^−1^) was calculated as a function of the CO_2_ desired partial pressure (pCO_2_ in %), the CO_2_ ambient partial pressure was at 0.04% and the gas mix flow rate was 15 l/min. The exposure to elevated CO_2_ was gradual and consisted of a first exposure to 800 µatm for 2 days, then 1100 µatm for 4 days, to then finally reach the target value of 1500 µatm for 7–10 days (depending on the experiment, see next section). The pH (NBS) was measured daily in randomly chosen tanks from both control and treatment groups using a WP-91 waterproof pH meter (TPS) and a Seven2GO pH meter (Mettler Toledo) calibrated every day with TPS 4.01, 7 and 10 calibration buffers. The total alkalinity (TA) was measured weekly by equivalent point titration using a G20S titrator (Mettler Toledo) with TA batch 155 as reference material for oceanic CO_2_ measurements (Scripps Institution of Oceanography). Using the titrator’s measurements, pCO_2_ values were calculated using the CO2_SYS_ software with K1, K2 constants from Mehrbach *et al*. 1973 refit by Dickson and Millero [47] and NBS pH scale [48]. Following the recommendations of the National Resource for *Aplysia*, temperature was set at 16°C using HC-1000A chillers (Hailea) and measured daily in randomly chosen tanks from both control and treatment groups using a WP-91 waterproof thermometer (TPS) and a Seven2GO thermometer (Mettler Tolder). Salinity was monitored daily using a portable refractometer. Weekly measurements of nitrate in randomly chosen tanks from both control and treatment groups were done using a HI97728 nitrate photometer (Hanna Instruments) to ensure good water quality. Tanks were cleaned every day by siphoning waste from the bottom of the tank. The animals were kept under a 12/12 h light–dark cycle. *Aplysia* were fed *Agardhiella subulata* algae every 3 days as instructed by the National Resource for *Aplysia*.

Across all tanks, the salinity was at 32 ± 1.6‰ and the temperature was at 15.5 ± 0.6°C for the whole experimental duration (electronic supplementary material, table S1). The elevated pCO_2_ conditions over the final 7–10 days of experiment were 1493.5 ± 82.3 µatm, whereas the control pCO_2_ was 679.9 ± 73.9 µatm throughout the experiment (electronic supplementary material, figure S2), consistent with previous observations of Hong Kong sea surface pCO_2_ values [49,50] and within the range of current levels experienced in the Californian coast [51,52], *A. californica*’s natural habitat [53], notably owing to the presence of upwelling [54,55]. pCO_2_ was significantly different between treatment groups (Wilcoxon rank sum test, *p*‐value < 0.001). Additionally, there was no effect of the rearing system identity on pCO_2_ within the ‘treatment’ group (ANOVA, *p*‐value = 0.628).

### 2.2. Behavioural assays

All behavioural assays were performed by video recording using a Canon EOS M50 camera. To test the innate reflex response to TWR after elevated CO_2_ exposure of 7 days, animals reared either in control (*n* = 14) or elevated CO_2_ conditions (pCO_2_ approx. 1500 µatm; *n* = 16) were transferred and acclimatized for 30 min in individual behavioural experimental tanks (12 × 18 × 2.5 cm) filled with seawater of their respective CO_2_ conditions. The TWR was then elicited three times per animal, by pressing onto the tail with a 20G gauge needle (see electronic supplementary material) and with each assay separated by 10 min (figure 1*a*), as performed in previous studies investigating the TWR [36,38]. Behavioural tanks were flushed between individuals. The water used originated from the individuals’ rearing regimes and was not further adjusted during the behavioural assays, as behavioural impairments in high pCO_2_ acclimatized animals were shown to be unaltered by experimental test water [56].

To test if learning is impaired under OA conditions, *Aplysia* either reared in control (*n* = 18) or elevated CO_2_ conditions (*n* = 17) received a habituation training after a 10 days exposure in their rearing tanks (figure 1*b*). A period of 30 min for acclimatization to the experimental set-up was given to individuals, and then the pre-training response was assessed three times, the same way as for the innate experiment, with a resting period of 5 min each. Training was then provided by delivering 30 repeated tail stimuli, with 30 s intervals between. The post-training TWR response was assessed 20, 25 and 30 min after training in the same way as pre-training. This protocol is the same as that of another previous study investigating habituation of the TWR [57].

To test if GABAergic neurotransmission is involved in observed behavioural impairments, *Aplysia* reared under near-future CO_2_ conditions (pCO_2_ approx. 1500 µatm) for 7 days were either administered the GABA_A_ receptor antagonist gabazine right after transfer to the behavioural tank (4 mg l^−1^ of SR-95531, Sigma; *n* = 14) or control water solution from their rearing tank (*n* = 16; figure 1*c*). The administration route was dissolution through the experimental tank water. The TWR behaviour was recorded after a 30 min acclimatization period similar to the innate experiment.

### 2.3. Behaviour statistical analyses

The reflex response was measured from video recordings in terms of duration by an observer blind to experimental conditions (electronic supplementary material, table S2). Measurements were discarded either if the tail was not clearly visible after retraction, or if the TWR triggered inking, or if the reflex was a siphon withdrawal reflex. Mean values were discarded if they were flagged as outliers with the Grubb’s test. Mean durations were compared between control and treatment groups for each experiment using the Student’s *t*‐test if the data were normally distributed, or else the Wilcoxon rank sum test when the data were not normally distributed. All statistical analyses were performed in R [58].

### 2.4. RNA sequencing and gene expression analysis

All individuals tested served for collection of RNA from the pleural–pedal ganglia, the nervous tissue part known to be in control of the TWR’s circuitry with sensory neurons located in the pleural ganglia and motoneurons found in the pedal ganglia [34,59–61]. The animals were dissected immediately following their behavioural assay and for each animal the pleural–pedal ganglia were snap frozen in liquid nitrogen and kept at −80°C until RNA extraction. The pedal–pleural ganglia were then homogenized using sterile silicon beads for 1 min at the highest frequency (300 Hz) in a Tissue Lyzer (Qiagen) and RNA was extracted following the TRIzol™ Plus RNA Purification Kit (Thermo Fisher). The RNA concentration and quality of some samples were measured using TapeStation (Agilent) to ensure good sample quality (RINe > 7). Samples were sequenced at 150 bp paired end on an Illumina NovaSeq at the Centre for PanorOmic Sciences (CPOS) of the University of Hong Kong. After sample sequencing, raw sequence data (on average 38.5 ± 3.9 M reads per sample; electronic supplementary material, table S3) were trimmed off adapters and filtered based on read quality using Trimmomatic [62]. Trimmomatic was run using the following parameters: ‘ILLUMINACLIP: all_PE.fa:2:30:10:8:TRUE LEADING:4 TRAILING:3 SLIDINGWINDOW:4:20 MINLEN:30’. Then, the filtered data (on average 36.5 ± 3.7 M reads per sample; electronic supplementary material, table S3) was mapped against the reference genome AplCal3.0 and its RefSeq annotation available for *Aplysia* [63], using the program HISAT2 [64].

Differential expression (DE) analyses were led using DESeq2 v. 1.38 [65] to investigate which genes were differentially expressed between *Aplysia* reared either in control or elevated pCO_2_. A likelihood ratio test (LRT) was used to ensure that neither the system nor the tank identity were factors of influence on gene DE. Then, a Wald test was performed to identify DE genes depending on the factor of interest. The design formula of innate and learning experiments (‘~pCO_2_’) allowed pairwise comparisons of groups depending on the two pCO_2_ levels, whereas the design formula of the GABA experiment (‘~Gabazine’) allowed pairwise comparisons between *Aplysia* either exposed to gabazine or control water, all at elevated pCO_2_. Differentially expressed genes with a baseMean under 10 and/or an absolute value of log2foldchange inferior to 0.3 were discarded to ensure that DE was not an artefact of low counts, and to increase stringency. Additionally, to identify which genes have their expression patterns possibly mediated by either the TWR response, CO_2_ and/or habituation training, and could in turn play a role in the behavioural change of *Aplysia*, a weighted gene co-expression network analysis (WGCNA) was performed using the WGCNA v. 1.72.1 package [66]. Mean TWR duration of each individual and final target pCO_2_ were provided as trait data, as well as binary encoded information regarding their habituation training status (‘0’ = naive, ‘1’ = trained) and gabazine exposure (‘0’ = not exposed to gabazine; ‘1’ = exposed to gabazine). The following parameters were used to build the network: power = 7 (with *R* ^2^ > 0.90), TOMType = ‘signed’, minModuleSize = 30, reassignThreshold = 0, mergeCutHeight = 0.25, verbose = 3. Clusters of genes whose expression patterns were correlated with either pCO_2_ and/or habituation training along with TWR duration, or with TWR duration alone were identified. For significant DE genes and for genes highlighted by the WGCNA analysis, functional enrichment analyses were performed using OmicsBox v. 1.4.11 (Fisher’s exact test). The gene ontology (GO) annotations used for the enrichment analysis were retrieved from BioMart in OmicsBox, using the ‘Database of genes from NCBI RefSeq genomes IDs’. The GO terms with an FDR-adjusted *p*-value below the 0.05 threshold were considered enriched among the DE genes, and the list of GO terms was reduced to its most specific. Uncharacterized loci highlighted by the gene expression analyses were searched in Rapid Ensembl release 109 [67] in the *A. californica* genome AplCal3.0 (assembly GCA_000002075.2) to predict their putative function.

## 3. Results

### 3.1. Behavioural response to ocean acidification during tail withdrawal reflex

**Figure 2.**
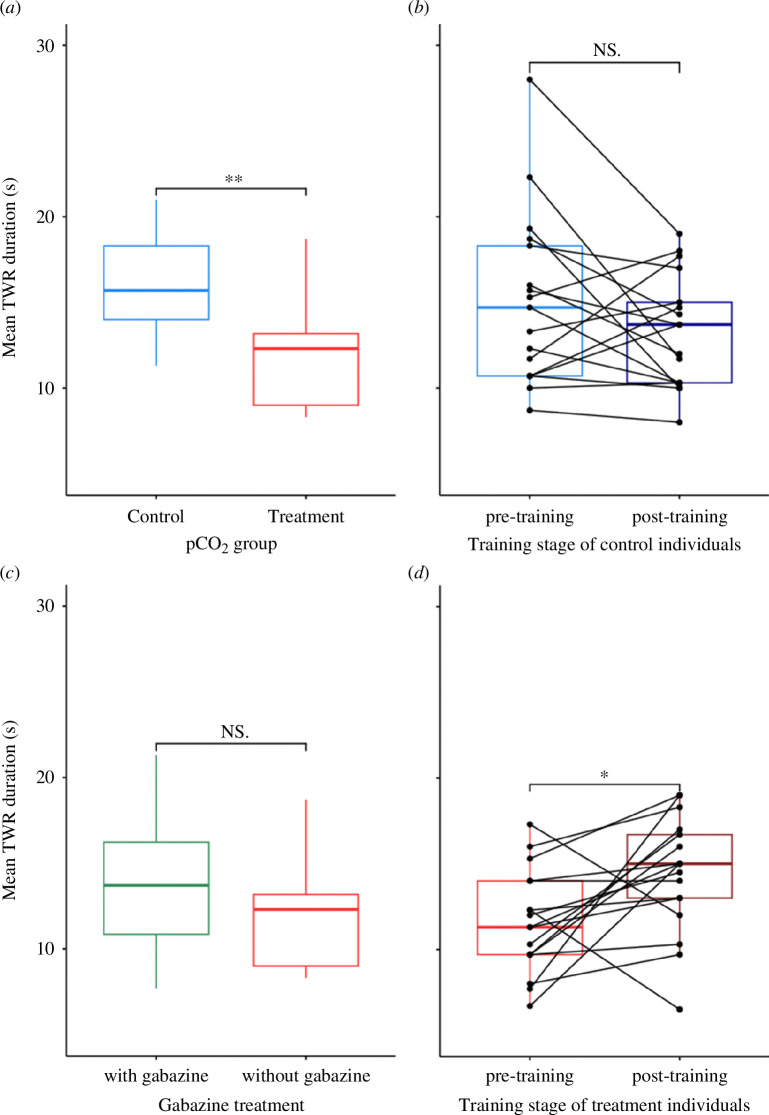
Mean TWR duration (s) of *Aplysia* in the innate experiment (*a*) as a function of their pCO_2_ conditions (control approx. 680 µatm or treatment approx. 1500 µatm), in the GABA experiment as a function of their gabazine exposure (with or without, (*c*)) and in the habituation experiment (*b,d*) reared at control pCO_2_ (*b*) and at elevated pCO_2_ (*d*), before (pre-training) and after (post-training) habituation training; stars (** or *) indicate the level of significant difference between the mean values whereas ‘NS.’ indicates the absence of significant difference between the mean values.

In the innate experiment, *Aplysia* exposed to elevated CO_2_ conditions relaxed their tail after withdrawal 29% faster with 12 ± 5 s (mean ± s.d.) to relax, which was significantly different from control condition which relaxed after 17 ± 6 s (Student’s *t*‐test, *p*‐value < 0.001; figure 2*a*). In the habituation experiment, we saw the same significant effect of CO_2_ exposure on TWR duration before habituation training (Wilcoxon rank sum test, *p*‐value = 0.0216; electronic supplementary material, figure S4), as *Aplysia* exposed to elevated pCO_2_ before conditioning took on average 12 ± 5 s to relax in elevated CO_2_ but 15 ± 7 s in control condition.

Habituation training caused *Aplysia* to relax their tail in 13 ± 3 s for control conditions and 14 ± 6 s for elevated pCO_2_ conditions. While it was not significantly different to pre-training for control individuals that relaxed their tail in 15 ± 5 s (Wilcoxon rank sum test, *p*‐value = 0.279; figure 2*b*), habituation training surprisingly increased TWR duration for all but two individuals exposed to elevated CO_2_ (*p*‐value = 0.0238; figure 2*d*). Elevated CO_2_ caused the tail to relax approximately 17% slower after conditioning. Finally, in the GABA experiment where all individuals were exposed to elevated CO_2_, contrary to predictions, there was no effect of gabazine on TWR duration (Student’s *t*‐test, *p*‐value = 0.1423; figure 2*c*). Individuals who were administered control seawater took on average 12 ± 5 s to relax from TWR whereas individuals who were administered gabazine relaxed after 14 ± 6 s.

### 3.2. Molecular response to ocean acidification during tail withdrawal reflex

**Figure 3.**
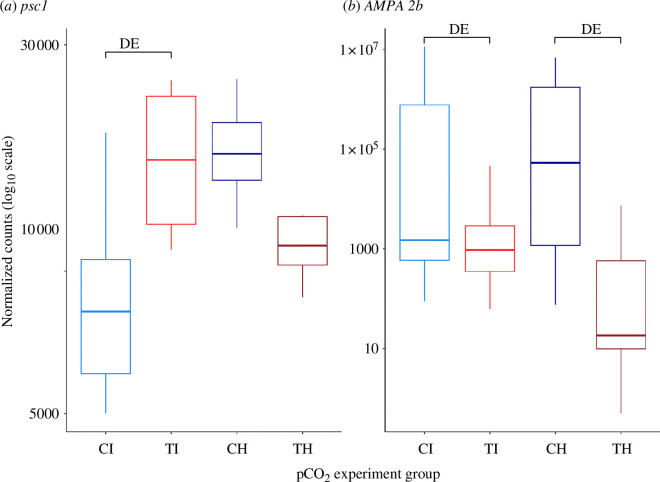
Normalized counts (log_10_) in all pCO_2_-experiment groups for the gene psc1 (*a*) and the AMPA 2b predicted coding gene (*b*); *Aplysia* were grouped as a function of the pCO_2_ they were reared at and the experiment they participated in: CI = control (approx. 680 µatm) Innate, TI = treatment (approx. 1500 µatm) Innate, CH = control habituation, TH = treatment habituation; ‘DE’ shows which pairwise comparison revealed the gene as differentially expressed.

**Figure 4.**
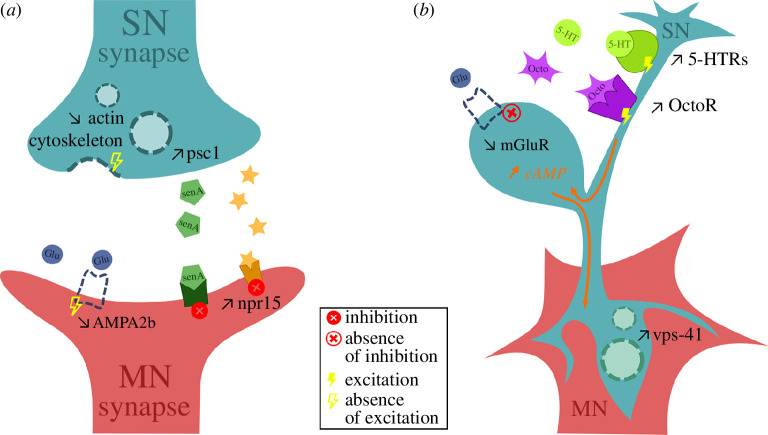
Molecular mechanisms in the TWR neural circuitry of *Aplysia* when exposed to elevated pCO_2_ (*a*) during innate elicitation of the reflex and (*b*) after habituation training of the reflex. SN = sensory neuron; MN = motoneuron; npr15 = neuropeptide 15 receptor; psc1 = pleural sensory cluster 1 gene, precursor of senA = sensorin A; Glu = glutamate; AMPA2b = AMPA 2b receptor gene; 5-HT = serotonin; 5-HTR = serotonin receptor; Octo = octopamine; OctoR = octopamine receptor; mGluR = metabotropic glutamate receptor; cAMP = cyclic adenosine monophosphate; *vps-41* = vacuolar protein sorting protein 41 gene.

Acidification provoked a large transcriptomic response in the TWR-mediating parts of the *Aplysia* nervous system, with a total of 1761 genes significantly differentially expressed between naive *Aplysia* exposed to different pCO_2_ levels, while 725 genes were differentially expressed among trained *Aplysia*. Of these, 248 genes were commonly differentially expressed owing to acidification. This general response to OA affected expression of genes involved in calcium ion (Ca^2+^) binding (electronic supplementary material, tables S4 and S5), notably of receptors of Ca^2+^ important to nervous system functioning. Calcium transport exhibited downregulation of calcyphosin-like gene possibly regulating ion transport and a trimeric intracellular cation channel gene (*tric-1B.2*), which facilitates the active intracellular release of Ca^2+^ (electronic supplementary material, tables S6 and S7). Additionally, calmodulins, which are calcium-binding messengers involved in calcium signal transduction and synaptic plasticity (electronic supplementary material, tables S6 and S7), and calcium-binding protocadherins involved in cell–cell interactions were also differentially expressed (electronic supplementary material, tables S6 and S7). Related to calcium signalling, OA also provoked DE of genes involved in excitatory neurotransmission with the upregulation of neuronal acetylcholine (ACh) receptor subunits, the cerebral peptide 2 precursor (*CP2PP*), the neuropeptide 15 receptor (*npr-15*), the neuropeptide CCHamide-1 receptor and an ionotropic glutamate receptor (figure 3*a*, electronic supplementary material, table S5). Furthermore, pCO_2_ levels positively correlated with an ACh receptor subunit precursor (electronic supplementary material, table S9). Another excitatory ionotropic glutamate receptor of the alpha-amino-3-hydroxy-5-methyl-4-isoxazolepropionic acid (AMPA) type was predicted for an uncharacterized locus (*LOC101845288*) in the genome, which had the highest expression level among all transcripts yet was significantly downregulated owing to acidification (figures 3*a* and 4*b*, electronic supplementary material, tables S6 and S7) in both naive and trained *Aplysia*. Acidification specifically had an effect on genes involved in serotonergic neurotransmission in naive *Aplysia* as the expression of two serotonin receptors with excitatory activity on neurons (*5-HT_2_* and *5-HT*_*4*_) but also one inhibitory serotonin receptor (*5-HT*_*1D*_), was expressed at higher levels at elevated pCO_2_ (figure 3*b*, and electronic supplementary material, tables S5 and S9). Another gene involved in inhibitory neurotransmission was the upregulation of BTB/POZ domain-containing protein *KCTD12*, which is a GABA_B_ receptor subunit (electronic supplementary material, table S6). Finally, acidification caused the upregulation of a calcium-binding inhibitory co-transmitter released by mechanosensory neurons, the precursor mRNA of sensorin-A (*psc1*; figure 3*a* and 4*a*; electronic supplementary material, table S6).

OA also affected expression of genes coding for components of the cytoskeletal network. Notably, the expression of actin-related genes was significantly positively correlated with mean TWR duration and negatively correlated with pCO_2_ (electronic supplementary material, figure S6; table S8), including actin-related protein (ARP) 2/3 complex subunits, ARPs, an F-actin-capping protein subunit, a myosin-10, a FLII actin remodelling protein, a plastin-1 and one actin protein (electronic supplementary material, table S9). Furthermore, genes involved in the actin–myosin interaction regulating cell morphology and cytoskeletal organization were differentially expressed, such as myosin light chains coding genes (electronic supplementary material, tables S6 and S7). Specifically in naive *Aplysia*, an actin-interacting protein, ARP 2/3 complex subunits and the PRKCA-binding protein (*pick1*) coding genes, which interact to regulate excitatory synaptic plasticity, were downregulated (electronic supplementary material, table S6). Other genes associated with cytoskeletal motion and intracellular transport were downregulated at elevated pCO_2_, such as genes coding for tubulin chains as well as the centrin-3 and EF-hand domain-containing protein-coding genes, which are involved in microtubule organization (electronic supplementary material, tables S6 and S7). Finally, genes interacting with dynein also involved in cytoskeletal motion were consistently affected by acidification, such as a parkin co-regulated gene homologue, a dynein assembly factor, dynein chains and dynein regulatory complex proteins (electronic supplementary material, tables S6 and S7).

Genes involved in several energy-related processes exhibited changes in expression with acidification. First, expression of genes involved in ATP metabolism through proton transmembrane transport, proton-transporting ATPase activity and acyltransferase activity (electronic supplementary material, table S4) were altered in naive *Aplysia* with the downregulation of ATP synthase, ATPase and ATP-citrate synthase and citrate synthase coding genes (electronic supplementary material, table S6). Biosynthesis of nucleic acids, which is energetically expensive, was downregulated (electronic supplementary material, table S4) and expression of genes involved in protein metabolism was affected by acidification through the downregulation of all genes involved in alpha-amino acid synthesis (electronic supplementary material, tables S4, S6 and S7) tRNA ligases, initiation factor subunits and elongation factors (electronic supplementary material, tables S6 and S7) in both naive and trained *Aplysia*. Genes involved in protein synthesis itself had DE, such as ribosomal protein-coding genes (electronic supplementary material, table S6) as well as the expression of post-translational modification glycosylation genes like downregulated protein glycosyltransferase subunits (electronic supplementary material, tables S6 and S7). Finally, downregulation of protein disulfide-isomerase coding genes (electronic supplementary material, table S6) suggested an impact on unfolded protein binding in naive *Aplysia* exposed to elevated pCO_2_ (electronic supplementary material, table S4).

Acidification triggered changes in the expression of cellular stress response genes. Heat shock proteins 70 (B2 subfamily) were upregulated in *Aplysia* exposed to elevated pCO_2_ (electronic supplementary material, tables S6 and S7) and genes with antioxidant activity (electronic supplementary material, table S4) were downregulated such as glutathione S-transferases, a glutathione reductase, a carbonyl reductase [NADPH] 1 isoforms, glutaredoxin and peroxiredoxins (electronic supplementary material, tables S6 and S7).

Habituation training revealed an effect of acidification on learning experiences in the nervous system of *Aplysia*. After *Aplysia* received habituation training, exposure to elevated pCO_2_ did not alter expression of genes involved in cell signalling and organization as seen in untrained *Aplysia*. Interestingly, the precursor mRNA of sensorin-A (*psc1*) was upregulated owing to acidification in naive *Aplysia,* but trained *Aplysia* had elevated levels of this gene no matter the pCO_2_ (figures 3*a* and 4*a*; electronic supplementary material, table S6), suggesting that overexpression of *psc1* could also be achieved through habituation training. Similarly, only in naive *Aplysia*, acidification provoked changes in the expression of genes involved in cell signalling processes mediated by GTP binding and GTPase activity (electronic supplementary material, table S4), with downregulation of G-proteins involved in vesicular traffic such as ADP ribosylation factor (ARF), ARF-binding protein (*GGA1*) and two ADP-ribosylation factor-like (ARL) proteins (electronic supplementary material, table S6), which were not altered by OA in trained *Aplysia*. Expression of genes involved in further processes changed for naive *Aplysia* with OA, but not in trained *Aplysia* despite the elevated CO_2_ levels notably regarding intracellular transport functions, notably vesicle-mediated and protein transport (figure 3*a*, electronic supplementary material, table S4). Expression in genes coding for vacuolar protein sorting-associated proteins, YIPF proteins, coatomer subunits and exocyst complex components, as well as a vesicle-associated membrane protein (VAMP)/synaptobrevin-binding protein, a synaptobrevin and a synaptobrevin homologue, downregulated for naive *Aplysia*, are at control levels in trained *Aplysia* (electronic supplementary material, table S6). Finally, otoferlin, involved in the Ca^2+^-triggered synaptic vesicle-plasma membrane fusion and in the control of neurotransmitter release at output synapses, was upregulated only in naive *Aplysia* but not in trained OA *Aplysia* (electronic supplementary material, table S6). Further evidence that habituation training and pCO_2_ affect genes involved in cell signalling differently was found as co-expressed genes positively correlated with pCO_2_ but negatively correlated with habituation training participated in GTPase mediated signalling (electronic supplementary material, table S8) such as four guanine nucleotide exchange factor coding genes and two genes coding for dedicators of cytokinesis (electronic supplementary material, table S9). Furthermore, genes of the thioredoxin antioxidant system and further antioxidant enzymes (glutathione peroxidase and superoxide dismutase) were at control levels for trained *Aplysia* but downregulated in naive *Aplysia* (electronic supplementary material, table S6) and these genes were also positively correlated with mean TWR duration and habituation training, but negatively correlated with pCO_2_ (electronic supplementary material, figure S6; electronic supplementary material, table S9). Overall, genes involved in cell signalling and cellular stress response whose expression were altered by acidification in naive *Aplysia* were not differentially expressed in trained *Aplysia*, indicating a possible ‘cancelling/antagonistic’ effect of habituation training on gene expression under acidification.

Habituation training, however, also caused specific effects associated with acidification in the nervous system of *Aplysia*. Signalling and vesicle transport genes were affected by the pCO_2_ level in their expression levels, but the genes involved in these functions were different after they experienced habituation training. For instance, regucalcin, which is a suppressor protein of cell signalling, was downregulated by acidification only in trained *Aplysia* (electronic supplementary material, table S7). Moreover, five genes involved in cellular transport were upregulated with elevated pCO_2_ only in trained *Aplysia*, notably two *vps-41* isoforms, one VAMP coding gene, one *VTI1B* homologue and one gene coding for an ARL protein 6-interacting protein (figure 3*b*, electronic supplementary material, table S7). Genes involved in GTP-mediated signal transduction were also differently affected by pCO_2_ after habituation training, as the small GTPase *Rap*, a Ras-related protein (*Rab-21*) involved in membrane trafficking, the *IQGAP1* gene regulating the dynamics of the actin cytoskeleton and the Ras Guanine nucleotide exchange factor (GEF) 1B were all upregulated in trained *Aplysia* (electronic supplementary material, table S7). Furthermore, genes involved in neuromodulation were downregulated at elevated pCO_2_ only in trained *Aplysia*, such as two metabotropic glutamate receptors involved in the regulation of neurotransmitter release notably by preventing cAMP production (figure 3*b*, electronic supplementary material, table S7). Further cAMP-responsive pathway genes including CREB-regulated transcription coactivator and CREB3 regulatory factor were upregulated specifically after habituation training (electronic supplementary material, table S7), and the CREB3 regulatory factor was also positively correlated with both pCO_2_ and habituation training, alongside the cAMP response element-binding protein (CREB) coding gene (electronic supplementary material, table S9). Both pCO_2_ levels and habituation training also positively correlated with the expression of serotonin and octopamine receptors involved in neuromodulation (figure 3*b*, electronic supplementary material, table S9). Finally, genes involved in oxidoreduction and cellular stress response were differentially expressed depending on pCO_2_ only in trained *Aplysia* such as the cytochrome *b*, the cytochrome *c* oxidase and nitric oxide synthase coding genes (electronic supplementary material, table S7). Therefore, habituation training triggers molecular changes specifically when individuals are experiencing acidification, such as modified expression of genes performing cell signalling, neuromodulation and oxidoreduction functions.

The potential role of GABAergic neurotransmission in the observed behavioural impairments owing to acidification was investigated by administering gabazine to *Aplysia* reared under near-future CO_2_ conditions. Only 20 genes were differentially expressed owing to the administration of gabazine in comparison with control water (electronic supplementary material, table S10). All except one (uncharacterized) were upregulated in *Aplysia* exposed to gabazine. They coded for hydrolases, proteases, kinases, a junction-mediating and regulatory protein (JMY), a cytochrome P450 2D26-like protein and a betaine–homocysteine S-methyltransferase 1 (electronic supplementary material, table S10).

## 4. Discussion

Our study connected the behavioural impairment caused by OA in *A. californica* with the molecular changes occurring in the relevant part of the nervous system and thereby pinpointing the underlying mechanisms of OA-driven behavioural alterations. Although the ambient pCO_2_ we used for our control group (approx. 680 μatm) was more elevated than the global average current atmospheric pCO_2_ (approx. 450 μatm), it is representative of what *A. californica* experiences in its natural habitat, the Californian coast [53] because the strong upwelling associated with the California Current causes more variable pCO_2_ levels, reaching over 700 μatm [51,55]. Acidification significantly decreased TWR duration consistent with a previous study [36] and provoked large changes in gene expression inside the TWR mediating parts of the nervous system, the pleural–pedal ganglia. Several of those observed changes constitute possible molecular mechanisms causing the behavioural impairment, such as the upregulation of the peptide sensorin-A (*psc1*) coding gene. As *psc1* is an inhibitory transmitter in *Aplysia* mechanosensory neurons [68], CO_2_-mediated increases in sensorin-A mRNA expression can cause downstream neurons of the circuit to be inhibited, therefore not transmitting the stimulus information to the end of the reflex circuitry which is responsible for prolonged contraction. Hence, by leveraging the intricate knowledge of the TWR circuit in the simple neuro system of *Aplysia* we are able to connect the behavioural impairment to underlying mechanisms.

Further neuronal signalling inhibition may be at play, however, as we find a neuropeptide receptor (*npr-15*) and an AMPA receptor changed expression levels when exposed to elevated CO_2_. These alterations could lead to an increase in inhibitory response, such as seen in an octopaminergic manner for *Caenorhabditis elegans* in the case of *npr-15* [69] with increased inhibition of neurons inside the reflex circuitry. Furthermore, reduced glutamatergic excitatory transmission via downregulation of the AMPA receptor may lead to decreased neuron excitability in *Aplysia* [38,70], resulting in less firing of motoneurons for prolonged foot muscle contraction. DE of receptors involved in neurotransmission can therefore lead to the decreased TWR duration caused by acidification.

Apart from neurotransmission, expression of genes involved in cellular organization and signalling is altered by elevated CO_2_ exposure and could be involved in the decreased TWR duration, notably genes involved in actin dynamics were correlated with TWR duration. The actin cytoskeleton regulates synaptic areas morphology and synaptic vesicle pools [71,72], and it is sensitive to OA in other molluscs [73,74]. In the case of *Aplysia*, synaptic vesicle delivery could be affected through the observed reduced expression of actin-related genes involved in the mobilization of synaptic vesicles. As a result, this reduced vesicle activity could in fact inhibit neurotransmission and decrease the reflex response and duration. Furthermore, other cytoskeletal motors, such as tubulin, were also downregulated by elevated pCO_2_. This same pattern has been found in tubeworm and oyster larvae [75,76]. Evidence of changes in cytoskeleton components during exposure to elevated pCO_2_ suggests that acidification may not only interfere with normal vesicle functioning at the synapse, causing behavioural impairment, but might also alter numerous other functions performed by the cytoskeleton. For example, in blood clams changes in the cytoskeleton owing to acidification may have implications on the immune response because cytoskeletal components perform phagocytosis [77]. Hence, cytoskeletal changes may reduce synaptic mobilization, which is a further potential mechanism for TWR changes under acidification conditions, but modified cytoskeleton organization could also cause other downstream effects at the whole organism level.

The decreased synaptic activity among neurons of the reflex circuitry with acidification may have led to compensatory mechanisms through DE of genes involved in neurotransmission. One potential compensation is the upregulation of acetylcholine receptors in *Aplysia* pedal–pleural ganglia, also observed in pteropod nervous systems under OA [78,79]. The upregulation of neuronal acetylcholine could increase Ca^2+^ levels and intensify calcium-mediated exocytosis of synaptic vesicles, which can be an alternative way of restoring neurotransmitter delivery impaired by the downregulation of genes involved in synaptic mobilization in *Aplysia*. Additionally, with otoferlin placing synaptic vesicles near calcium channels to allow fast exocytosis of neurotransmitters [80], the observed upregulation of otoferlin in *Aplysia* at elevated pCO_2_ could also facilitate Ca^2+^-mediated release of synaptic vesicles from the sensory neurons and restore a flow of neurotransmitters in the synaptic area. Furthermore, the upregulation of genes involved in the recycling of synaptic vesicles could be a compensatory mechanism in *Aplysia* when faced with acidification. Maintenance of neurotransmission by recycling existing receptors could counteract decreased gene expression of such receptors. For example, the upregulation of the huntingtin-interacting protein and AP180 coding genes in *Aplysia* neurons at elevated pCO_2_ could maintain neurotransmission by enhancing the recycling of already existing AMPA receptors [81,82], despite the downregulation of the AMPA receptor coding gene that would limit its neobiosynthesis. Finally, increased production of neuropeptides, such as those derived from the cerebral peptide 2 precursor (*CP2PP*) could also be a way to compensate for impaired neurotransmission. *CP2PP* is a precursor to 10 bioactive neuropeptides in *Aplysia*, among which some act on the foot muscle [83,84]; therefore, its upregulation could lead to increased production of neuropeptides which could activate tail motoneurons. Overall, *Aplysia* shows signs of transcriptional reprogramming to compensate for reduced neurotransmission when exposed to acidification in the nervous system.

The molecular response to acidification in the pedal–pleural ganglia also comprised several important functions such as ATP, nucleic acid and protein metabolism. Downregulation of metabolism genes in *Aplysia* could be a sign of metabolic suppression as commonly seen in other invertebrates when exposed to elevated pCO_2_ [85–88], implying that downregulation of oxidative metabolism genes in sensitive organisms might compromise the cellular stress response [89]. However, in *Aplysia*, the downregulation of oxidative metabolism genes under OA is not sufficient to imply that the nervous system may be more susceptible to cellular stress, because the upregulation of heat shock protein genes 70 was also observed. This type of heat shock protein is expressed when cellular protection is required, but notably not restricted to oxidative stress conditions [90]. Their upregulation in *Aplysia* to protect cells against OA-mediated stress has also been documented in Sydney rock oysters [91]. Hence, instead of metabolic suppression, the metabolic downregulation may rather point to reallocation of energy towards homeostasis. This has been suggested as a cellular strategy to redirect the energy in the most effective way possible towards immediate essential processes at the expense of other functions [88]. *Aplysia* exposed to 1200 µatm of pCO_2_ are able to maintain their haemolymph pH_e_ levels similar to that of control by accumulating HCO_3_^−^ [36]. It is therefore possible that the downregulation of genes involved in expensive processes is a means to ensure that sufficient resources are allocated to the uptake of HCO_3_^−^ inside the haemolymph to maintain acid-base homeostasis at the whole organism level [92–94].

When *Aplysia* were trained under OA, contrary to our expectations, the TWR duration increased. Increased duration is consistent with short-term sensitization rather than habituation [44]. The cellular mechanism underlying short-term sensitization training in *Aplysia* consists of the activation of serotonergic neurons, which act on the sensory neurons of the reflex circuitry by increasing cAMP intracellular levels [95,96]. Hence, increased expression of serotonin and octopamine receptors in the neural circuitry at elevated CO_2_ following habituation training could cause a sensitization and produce the increased TWR duration. Furthermore, sensitization-like responses may also be caused by the alteration of metabotropic glutamate receptors. A downregulation of *MGR2* activation, as exhibited in trained *Aplysia* with OA, could increase production of cAMP in the presynaptic area following habituation training and produce changes in the nervous system consistent with sensitization [97]. Trained *Aplysia* exposed to elevated pCO_2_ resulted in specific molecular changes related to the habituation training process, for example of *vps-41* coding gene expression. Expression of circular mRNA of *vps-41* improves synaptic plasticity and learning functions by acting as a ‘sponge’ delivering miRNAs to regulate the transcriptome [98,99]. Its upregulation under OA and after habituation training could therefore facilitate synaptic changes in the reflex circuitry. Upregulation of *vps-41* could strengthen synaptic connections between sensory neurons and motoneurons through decreased presynaptic transmitter release [100], leading to a sensitization-like behavioural response after elicitation of the reflex. Overall, habituation training while experiencing OA provokes molecular changes that could act on the nervous system in a similar way to that of a sensitization experience in normal conditions, hence modulating the behaviour differently under the influence of changes pH conditions.

*Aplysia* exposed to elevated CO_2_ and administered gabazine surprisingly did not show restoration of TWR duration consistent with that of control individuals and few changes were observed on the molecular level. Previous studies in molluscs have demonstrated that gabazine could restore impaired behaviours such as burrowing and predator escape [13,101], therefore in invertebrates, gabazine can be an antagonist of GABA_A_ receptors. However, its pharmacological effect on *Aplysia* reared in control conditions is not known. Hence, it is possible that gabazine could produce a shorter TWR duration irrespective of the pCO_2_ by blocking ion flow even under normal conditions. Therefore, it is difficult to pinpoint the specific role of GABA_A_ receptors in the TWR impairment since the action of gabazine may have effects on its own, potentially producing behavioural alterations [102]. Furthermore, other non-specific GABA antagonists like picrotoxin revealed an effect on CO_2_-sensitive behaviours in molluscs [32], suggesting that other receptors than GABA_A_ may be impaired with OA. Therefore, we cannot conclude that the behavioural change with OA is not owing to impaired GABA neurotransmission. Alternatively, it is possible that other ligand-gated ion channels (LGICs) performing neurotransmission could have their function altered by the acid–base regulation response, such as glutamate or acetylcholine-gated chloride channels which are affected by OA in *Aplysia* [31]. Hence, our results are only suggestive that different mechanisms than impaired GABA_A_ function could be responsible for TWR impairment in *Aplysia*.

Overall, our study reveals links between behavioural alterations and molecular changes occurring in the model neurosystem of *Aplysia* as it is experiencing OA. Our findings show that the TWR and its modulation through learning is impaired during OA through alterations in neurotransmission between mechanosensory neurons and neurons downstream of the reflex circuitry. Gabazine did not restore TWR duration like in other invertebrates, although it cannot disprove impaired GABAergic neurotransmission to be responsible for impaired behaviour. Exposure to elevated CO_2_ may overall lead to complex changes within the nervous system of *Aplysia* affecting even a simple behaviour, with its controlling neural circuit being sensitive to various molecular effects of elevated CO_2_. Such variability in molecular modifications may explain differences in the behavioural effects of elevated CO_2_ across individuals and species. By leveraging the simple nervous system of *Aplysia*, we show direct links between near-future predicted ocean conditions and the molecular processes occurring in neurological systems that control for animal behaviour.

## Data Availability

The raw sequencing data can be found in BioProject PRJNA1031344. The reviewer link to the data is. Electronic supplementary material is available online at [103].
